# A Systematic Review of Psychometric Properties of Knee-Related Outcome Measures Translated, Cross-Culturally Adapted, and Validated in Arabic Language

**DOI:** 10.3390/healthcare10091631

**Published:** 2022-08-26

**Authors:** Mahamed Ateef, Mazen Alqahtani, Msaad Alzhrani, Abdulaziz A. Alkathiry, Ahmad Alanazi, Shady Abdullah Alshewaier

**Affiliations:** Department of Physical Therapy and Health Rehabilitation, College of Applied Medical Sciences, Majmaah University, Al Majmaah 11952, Saudi Arabia

**Keywords:** osteoarthritis (OA), knee, assessment tools, Arabic language

## Abstract

During the previous two decades, patient-reported outcome measures (PROMs) have been well tested, and the tools were validated in different languages across the globe. This systematic review aimed to identify the knee disease-specific outcome tools in Arabic and evaluate their methodological quality of psychometric properties of the most promising tools based on the COSMIN checklist and PRISMA guidelines. Articles published in English, from the inception of databases until the date of search (10 August 2022), were included. Articles without at least one psychometric property (reliability, validity, and responsiveness) evaluation, and articles other than in the English language, were excluded from the study. The key terms [“Arabic” AND “Knee” AND (“Questionnaire” OR “Scale”)] were used in three databases, i.e., PubMed, Scopus, and Web of Science (WoS) in the advanced search strategy. Key terms were either in the title or abstract for PubMed. Key words were in the topic (TS) for WoS. COSMIN (COnsensus-based Standards for the selection of health Measurement Instruments) risk of bias checklist was used to evaluate the methodological quality of psychometric properties of the Arabic knee-related outcome measures. A total of 99 articles were identified in PubMed, SCOPUS, and WoS. After passing inclusion and exclusion criteria, 20 articles describing 22 scales from five countries were included in this review. The instruments validated in the Arabic language are Western Ontario and McMaster Universities Osteoarthritis Index (WOMAC), knee injury and osteoarthritis outcome score (KOOS), knee outcome survey- activities of daily living scale (KOS-ADLS), Oxford knee score (OKS), anterior knee pain scale, osteoarthritis of knee and hip health-related quality of life (OAKHQoL) scale, Lysholm knee score (LKS), international documentation committee subjective knee form (IKDC), intermittent and constant osteoarthritis pain (ICOAP) questionnaire, Kujala patellofemoral pain scoring system (PFPSS), anterior knee pain scale (AKPS) and osteoarthritis quality of life questionnaire (OAQoL),. All were found to have good test-retest reliability (Intra Correlation Coefficient), internal consistency (Cronbach’s alpha), and construct validity (Visual Analog Scale, Short Form-12, RAND-36, etc.). Of 20 instruments available to assess self-reported knee symptoms and function, 12 were validated in the Saudi Arabian population. Among them, KOS-ADLS is the best PROM to be used in various knee conditions, followed by KOOS and WOMAC. The assessed methodological quality of evidence says that the knee Arabic PROMs are reliable instruments to evaluate knee symptoms/function.

## 1. Introduction

Knee pain is one of the most common musculoskeletal conditions, with every fifth individual aged 30 or over suffering from knee pain [1,2]. Age, female gender, and obesity are some risk factors for knee pain, including knee osteoarthritis (OA) [2,3,4]. More than 250 million people are affected globally, with increased years lived with disability [5,6]. A recent report from the Middle East and North African region highlights the 9.3% increase in the prevalence of knee osteoarthritis, from 5342.8/100,000 when compared to 1990 [7]. Among them, Saudi Arabia, Kuwait, and Iran, have the highest prevalence [7]. The most common reason for knee joint pain is knee OA. Both subjective and objective measures are used in knee OA assessment as measures to determine the disease progression and prognosis for [2,3,8,9] treatment effectiveness by orthopedic surgeons [10] and physical therapists [11].

When the patient visits the clinic for a knee injury or pain treatment, there are many ways to collect subjective information, such as face-to-face interaction, questionnaires/scales/scores, telephonic conversation, and narration from the patient’s attendant. Questionnaires/scales/scores are more reliable and reproduce the disease status or characteristics in a more comprehensive way, unlike other methods of interaction where patients may miss or give inadequate or inappropriate disease information to the clinician, which may not help the full recovery of the patient. In addition, the patient-reported outcome measure (PROM) allows patient assessment to help the clinician and therapist to set appropriate goals, depending on the individual. Several PROMs are available for assessing knee joint symptoms and various pathology-related outcomes [12,13,14,15,16], and most of them are developed in English, then translated into different languages [17]. However, a simple translation of the original version does not guarantee similar measurement properties as cultural context differences must also be considered [18,19].

Modern Arabic is the world’s third most common official language (27 states, mainly in Arab League countries situated in North Africa and Gulf Peninsula). Moreover, Arabic is the sacred language of Islam [20] and has been spoken by more than 300 million people. Patients may not be able to reproduce their disease symptoms due to illiteracy or inappropriate adaptability to the English language. Patients feel more comfortable reproducing their disease suffering when they use their mother tongue, rather than originally available English versions [17]. However, many words and meanings totally differ among Arab countries. Therefore, the translation and adaptability of original questionnaires into local Arabic are critical to obtaining comprehensive, subjective data from the local population to evaluate a specific disease. While cross-culturally translating and validating the questionnaires, certain knee constructs need to be validated to obtain and equalize original language outcomes [21].

However, a psychometrically validated questionnaire would provide better outcomes. Hence, this systematic review mandates identifying and quantifying the methodological quality of the tools adapted and used for better patient-reported outcomes in the Arabic population with knee OA. Therefore, the objective of this systematic review was to identify the translated and cross-culturally adapted knee disease-specific outcome measures in the Arabic language, and to evaluate the methodological quality of psychometric properties of the PROMs.

## 2. Materials and Methods

### 2.1. Registration and Protocol

The protocol of this systematic review has followed the PRISMA (Preferred Reporting Items for Systematic reviews and Meta-Analysis) guidelines [22] and registered with the International Prospective Register of Systematic Reviews (PROSPERO) PROSPERO, reg. No. CRD42020203456 Available from: https://www.crd.york.ac.uk/prospero/display_record.php?RecordID=203456&VersionID=1580292 (accessed on 13 August 2020). According to the registered protocol, we must complete the review by 31 August 2020, but due to unavoidable circumstances, we have extended our search and update until 10 August 2022.

### 2.2. Eligibility Criteria

Articles published in English, from the inception of databases until the date of search (10 August 2022), were included. Articles that focused on cross-cultural validation and psychometric analysis in the Arabic version of subjective knee outcome measures of various knee disease populations, such as OA knee, ACL, meniscus, patellofemoral knee, and post-surgical patients. Review articles, articles without at least one psychometric property (reliability, validity, and responsiveness) evaluation, and articles other than in the English language, were excluded from the study.

### 2.3. Information Sources

The search was done in three databases, namely MEDLINE/PubMed, SCOPUS, and Web of Science (WoS) search engines, from inception until 10 August 2022.

### 2.4. Search Strategy

Key terms used were [(“Questionnaire” OR “Scale”) AND “Knee” AND “Arabic”] in the advanced search option. Key terms were used in the ‘title/abstract’ section of PubMed and the ‘topic (TS)’ of WoS. 

### 2.5. Selection Process

Two reviewers (MAq and AAk) independently assessed the title and abstract of the studies, then full-text articles based on the inclusion and exclusion criteria. Cohen’s kappa coefficient calculated the strength and agreement between the authors [20]. Both reviewers discussed with the first author (MAt) cases of conflicts, and concluded consensus. MAz, SAs, Aal and Sal have reviewed the articles for final approval.

### 2.6. Data Collection Process

The cross-cultural adaptation process was followed by guidelines given by Beaton et al. [18], and the COSMIN guideline assessed measurement properties [21].

### 2.7. Data Items

COSMIN risk of bias checklist is a standardized and validated scoring tool with 10 boxes, two of which are on content validity, three on internal structures, and the remaining five on measurement properties of PROM. Each box was assessed by various standards (items) and each standard (item) was scored on a three points rating scale (i.e., from high-to-low: “+” = sufficient, “−” = insufficient, “?” = indeterminate) [23]. An overall score from the study’s methodological quality was determined by taking the lowest rating of the item in the box [21]. We did not report the content validity because it must be done in a lengthy, systematic way, and many PROMs had not mentioned the standards reported by Terwee et al [24]; instead, we used Beaton et al. stages of cross-cultural translation [18]. Cross-cultural translation consists of five stages: forward translation, synthesis, backward translation, expert review, and pilot study [18].

Structural validity, internal consistency, and cross-cultural validity, were covered under the internal structure. Five boxes were presented under other measurement properties: reliability, measurement error, criterion validity, hypothesis testing for construct validity, and responsiveness. Apart from assessing boxes by standards (items), we evaluated each box by updated criteria for good measurement properties [22]. Overall qualities of each PROM were determined through a modified GRADE approach (Grading of Recommendations Assessment, Development, and Evaluation) [25]: (1) risk of bias-the methodological quality of the studies; (2) inconsistency- unexplained inconsistency of results across studies; (3) imprecision -total sample size of the available studies; and (4) indirectness-evidence from different populations than the population of interest in the review [18] by combining scores of COSMIN (COnsensus-based Standards for the selection of health Measurement Instruments) [21]. During the quality assessment process, only measurement properties (boxes) reported in each PROM were evaluated, and other boxes were considered NR (not reported). Therefore, it was not necessary to include all properties (boxes).

### 2.8. Study Risk of Bias in Individual Studies

Any missing stages in the cross-cultural adaptation process, or missing boxes in the COSMIN risk of bias checklist, were considered risk bias for PROM. A country of origin other than Saudi Arabia was considered a risk bias because many words’ wordings and meaning differ from one Arab country to the other. The translation and adaptability of original questionnaires into local Arabic are critical to obtaining comprehensive, subjective data from the local population to evaluate a specific disease. Two independent reviewers assessed each included study manually and came to a consensus.

### 2.9. Effect Measures

Validity was assessed by correlation, internal consistency by Cronbach’s alpha; and reliability either by intraclass correlation coefficient (ICC) or by Pearson’s/Spearman correlation coefficient (r/ρ). Measurement error should be reported by the standard error of the mean (SEM), minimal detectable change (MDC), or by the area under the curve (AUC), and responsiveness should be reported by effect size (ES) or pre-post analysis.

### 2.10. Synthesis Methods

Reliability/validity is considered strong if ICC is 0.70 or more and if ‘r’ is 0.80 or more. Internal consistency is considered strong if Cronbach’s alpha is 0.70 or more. Effect size (ES) is classified into weak, moderate, and strong, by 0.2–0.49, 0.5–0.79, and 0.8 or more, respectively [24]. Convergent validity of 0.50 or more was considered an acceptable hypothesis for construct validity [18].

## 3. Results

### 3.1. Study Selection

**Figure 1:**
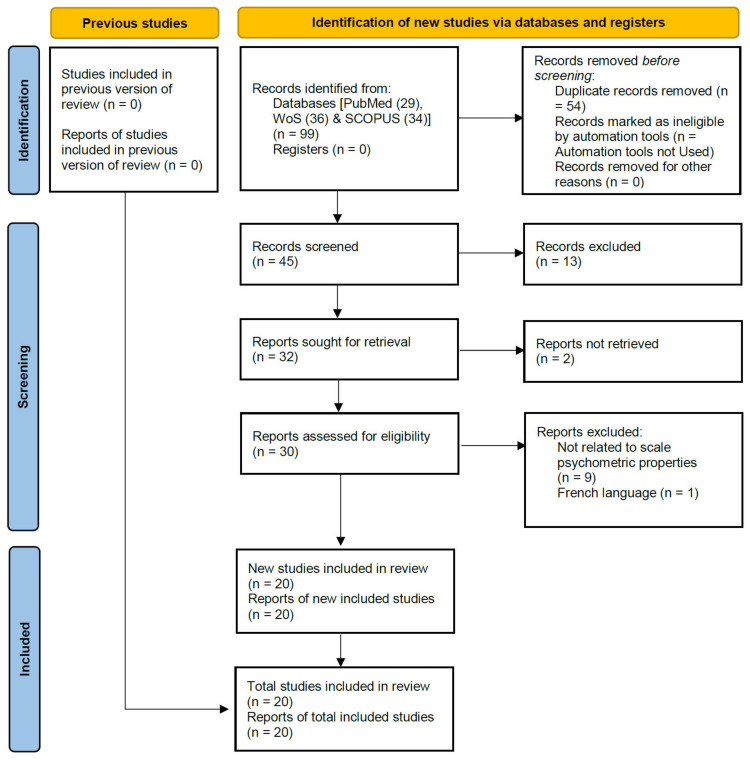
PRISMA 2020 statement highlighting the studies selected at each stage.

Entering key terms in search has resulted in a total of 99 hits in PubMed, Scopus, and Web of Science (WoS). Removing duplicates and adding one article from references of searched articles has resulted in 45 articles at stage II. After eliminating articles based on inclusion and exclusion criteria, we finally selected 20 articles for this review. Study selection is diagrammatically represented using the PRISMA flowchart in Figure 1.

### 3.2. Study Characteristics

A total of 20 articles were selected for this review; out of which 12 articles were from Saudi Arabia [1,25,26,27,28,29,30,31,32,33,34,35], two each from Morocco [36,37], Egypt [38,39], and Jordan [40,41], and one each from Tunisia [14], and Kuwait [42]. A total of 10 knee-related outcome measures were cross-culturally adapted, and their psychometric measurement properties were evaluated in the Arabic language. They were Western Ontario and McMaster universities osteoarthritis index (WOMAC) [1,14,37], knee injury and osteoarthritis outcome score (KOOS) [33,34,35,38,39,43] knee outcome survey- activities of daily living scale (KOS-ADLS) [30,42]. Oxford knee score [1], anterior knee pain scale [31], osteoarthritis of knee and hip health-related quality of life (OAKHQoL) scale [37], Kujala patellofemoral pain scoring system [40,41], intermittent and constant osteoarthritis pain questionnaire (ICOAP) [25], short version of anterior cruciate ligament-return to sport after injury scale (ACL-RSI) [26], and Tegner activity scale (TAS) [27]. WOMAC was validated in three different ways, i.e., reduced WOMAC [44], original WOMAC [37], and Sfax WOMAC [14], that were evaluated in three different nations, i.e., Saudi Arabia [1], Morocco [37], and Tunisia [14]. KOOS was validated in two different clinical populations, i.e., knee OA [43] and knee ligament injuries [39] from two different nations, i.e., Saudi Arabia [43] and Egypt [39]. KOS-ADLS was validated in two different nations, i.e., Saudi Arabia [29] and Kuwait [42], in different knee conditions (Table 1). All PROMs have at least two subscales, except one, i.e., Oxford knee score (OKS) [29,34,38]. OAKHQoL has a maximum of six subscales [36], followed by KOOS (five subscales) [33,35,39,43], WOMAC (two to three subscales) [14,28,37], and KOS-ADLS [30,42], anterior knee pain scale (two subscales each) [31,40]. The function subscale was present in all PROMs, followed by pain and symptom subscales (three PROMs each). Most of these tools were evaluated on knee osteoarthritis patients [14,28,29,35,36,37,38,43], apart from three on patellofemoral pain syndrome [31,40,41], anterior cruciate ligament/ligamentous injury, and meniscal injury [26,27,38,39]. Two studies used the population of various knee-related conditions to evaluate their tools [30,42] (Table 1).

The gap between the test and retest ranged from 24 h [14] to more than one week [34,36] and up to 15 days [38,40], with a median of 2–4 days [42]. All except one [14] used primarily self-report as an assessment method. (Table 1).

### 3.3. Risk of Bias within Studies

Among the 20 included studies, three [25,28,40] have not formed an expert review (stage IV) to examine or discuss back translation; two studies did not mention stage II [30,32]; three studies did not mention stage IV [14,25,40], and two studies did not mention stage V [32,40], respectively. All except two [26,36] used at least 10 patients at stage V (pilot study). Recall may be a source of bias in one study’s test-retest reliability [14]. Mode of administration was an interview based on one study [14,32]. The clinical population was not homogenous in three studies [30,38,42]. OAKHQoL, Kujala PFPSS, LKS, and IKDC were not validated in the Saudi Arabian population (Table 1). Four studies included only males [26,27,29,33], while one study included only females [35], and in another two studies, the majority (~90%) of the patients were females. Eight studies [14,36,37,38,39,40,41,42] were from countries other than Saudi Arabia, using local dialects in their PROM.

Internal consistency of individual subscales was not mentioned for KOS-ADLS [27,30,42] and anterior knee pain scale [31]. Evaluation of internal consistency was mentioned in the methodology, but values were not given in the results for reduced WOMAC [28]. Test-retest reliability of individual subscales was not given for anterior knee pain scale [31] and KOS-ADLS [42]. Measurement error was not reported for OAKHQoL, LKS, IKDC, Kujala PFPSS, ICOAP, and OAQoL [25,32,36,38,40,41]. Measurement error (only SEM, not MDC, or AUC) for subscales was not provided for anterior knee pain scale [31].

### 3.4. Results of Individual Studies

The minimal clinically important difference (MCID) was reported for four PROMs, i.e., reduced WOMAC, KOOS, Oxford knee score (all from Saudi Arabia), and KOS-ADLS (Kuwait). Subgroup analyses for age, sex, and joint involvement, were done for OAKHQoL [31]. During the cross-cultural validation process, Sfax WOMAC removed three questions from the function subscale based on the floor effect. (Table 2).

All studies evaluated at least three boxes/properties out of a possible eight boxes (range 3–5; median 4). All studies checked construct validity through hypothesis testing, followed by test-retest reliability (18 studies; one study not clear), internal consistency (16 studies; one study unclear), and measurement error (seven studies). None of the studies checked criterion validity, and one study each checked the cross-cultural validity and responsiveness (Table 2, Table 3 and Table 4). Table 3 shows good measurement properties of individual included studies. It shows enough results for internal consistency and test-retest reliability. There are intermediate results for construct validity and measurement error.

### 3.5. Results of Synthesis

Structural validity of PROM through factor analysis was done for KOS-ADLS [29], Sfax WOMAC [14], and KOOS [39]. Cronbach alpha value for internal consistency was greater than 0.8 for all subscales of included PROMs, except OAKHQoL’s social support and social function subscales (divergent items) [36]. Similarly, ICC values for test-retest reliability were greater than 0.8 in all subscales of reported PROMs, except OAKHQoL’s social support and social function subscales. All SEM, MDC, and AUC for measurement error were reported in only one study, i.e., KOS-ADLS [42]; four studies reported SEM and MDC for all subscales in their results, i.e., reduced WOMAC [28], KOOS [43], Oxford knee score [29], and KOOS-PF [33], two studies reported only SEM, i.e., anterior knee pain scale [31], KOOS [39]. Construct validity measured by convergent correlation was within an acceptable value of 0.5 or more, except five PROM subscales, i.e., WOMAC ADL subscale [37], OAKHQoL pain subscale [36], Sfax WOMAC function subscale [14], IKDC [38], and ICOAP [25]. Responsiveness was given for only one study, i.e., KOS-ADLS [42], with a strong ES of 1.12. Detailed quality criteria based on updated COSMIN guidelines for good measurement properties are tabulated in Table 5.

There were three studies each in WOMAC and KOOS, and two in KOS-ADLS. We applied a modified GRADE approach to these PROMs. KOOS had a ‘high’ grade for internal consistency property, followed by WOMAC (‘low’ grade) and KOS-ADLS (‘very low’ grade). KOS-ADLS had a ‘moderate’ grade for test-retest reliability property, followed by WOMAC (‘low’ grade) and KOOS (‘very low’ grade). WOMAC had a ‘high’ grade for measurement error property, followed by KOOS (‘moderate’ grade) and KOS-ADLS (‘low’ grade). All three PROMs had a ‘moderate’ grade for construct validity property. All PROMs were validated on more than 250 patients in at least two countries. KOOS was validated in two different clinical populations, apart from KOS-ADLS.

**Table 2:** Disease characteristics and instrument administration of the included study populations.

		Population			Disease Characteristics			Instrument Administration			
PROM *	Ref	N	Age Mean (SD, Range) yr.	Gender % Female	Disease	Disease Duration Means (SD) yr.	Disease Severity	Setting	Country	Language	Response Rate
Reduced WOMAC	Alghadir et al. 2016 [28]	140	Mean 52.95 SD 9.31 Range 40–80	53.7%	Knee OA		K/L 1 15.7% K/L 2 45.5% K/L 3 29.8% K/L 4 09.1%	Physiotherapy OP	Saudi Arabia	Arabic	86.43%
KOOS	Alfadhel et al. 2018 [43]	136	Mean 58.77 SD 9.1 Range	66.91%	Knee OA	Mean 5.91 SD 5.3	Mild 15% Moderate 33% Severe 52%	Physiotherapy Outpatient	Saudi Arabia	Arabic	89.71%
KOS-ADLS	Algarni et al. 2017 [30]	280	Mean 54.6 SD 10.5 Range	57.14%	Various Knee conditions (Knee OA 88.2%)		Knee OA 88.2% Patellofemoral syndrome 7.9% RA 3.9%	Outpatient Clinic	Saudi Arabia	Arabic	NR
Oxford Knee Score	Alghadir et al. 2017 [29]	97	Mean 57.55 SD 11.49 Range 40–80	0%	Knee OA			NR	Saudi Arabia	Arabic	100%
Anterior Knee Pain Scale	Alshehri et al. 2017 [31]	40	Mean 34.7 SD 9.3 Range	35%	Patellofemoral pain syndrome	Longer than 2 months		Hospital	Saudi Arabia	Arabic	NR
Moroccan WOMAC	Faik et al. 2008 [37]	71	Mean 56.83 SD 8.28 Range 36–84	94.4%	Knee OA	Mean 6.24 SD 5.04			Morocco	Arabic (Moroccan)	NR
Moroccan OAKHQoL	Serhier et al. 2012 [36]	135	Mean 56 SD 10 Range	89%	Knee and Hip OA			Clinic and Rehabilitation setting	Morocco	Arabic (Moroccan)	97%
Sfax Mod WOMAC	Guermazi et al. 2004 [14]	103	Mean 55.9 SD 7.67 Range 40–78	75.73%	Knee OA	Mean 4.0 SD 4.12	Mean K/L 2.74 SD 0.77 Range 1–4	NR	Tunisia	Arabic (North African dialect)	NR
KOOS	Almangoush et al. 2013 [39]	129	Mean 30.8 SD Range	23.3%	ACL, meniscal, and combined injury	7.2 months (Range 1–36 months)	ACL 38.0% Meniscal 27.9% Combined 34.1%	Knee centre	Egypt	Arabic	87%
KOS-ADLS	Bouzubar et al. 2018 [42]	108	Mean 44.3 SD 14.5 Range 19–71	48.1%	Various Knee clinical and post-surgical conditions		OA 34.9% PFPS 13.2% Ligament reconstruction 12.2% Arthroplasty 10.4%	Govt hospital and physiotherapy department	Kuwait	Arabic	4 weeks- 90.74%
Kujala PFPSS	Hamdan et al. 2019 [40]	97	Mean 43.34 SD 14.50 Range 40–80	69.1%	PFPS		Anterior knee pain	Orthopaedic surgery clinic	Jordan	Arabic	76.4%
LKS	Ahmed et al. 2019 [38]	100	ACL tear (Mean: 21.5; Range: 18–25), meniscus tear (Mean: 27.3; Range: 25–30) and OA (Mean: 50.7; Range: 40–70)	45%	ACL tear, meniscus tear, and knee OA	15 days Pre-OP, 1 day Pre-OP, and 6 months post-OP	ACL tear (*n* = 30), meniscus tear (*n* = 20) and knee OA (*n* = 50)	University Hospital	Egypt	Arabic	98%
OKS	Ahmed et al. 2019 [38]	100	ACL tear (Mean: 21.5; Range: 18-25), meniscus tear (Mean: 27.3; Range: 25–30) and OA (Mean: 50.7; Range: 40–70)	45%	ACL tear, meniscus tear, and knee OA	15 days Pre-OP, 1 day Pre-OP, and 6 months post-OP	ACL tear (*n* = 30), meniscus tear (*n* = 20) and knee OA (*n* = 50)	University hospital	Egypt	Arabic	95%
IKDC	Ahmed et al. 2019 [38]	100	ACL tear (Mean: 21.5; Range: 18–25), meniscus tear (Mean: 27.3; Range: 25–30) and OA (Mean: 50.7; Range: 40–70)	45%	ACL tear, meniscus tear, and knee OA	15 days Pre-OP, 1 day Pre-OP and 6 months post-OP	ACL tear (*n* = 30), meniscus tear (*n* = 20) and knee OA (*n* = 50)	University hospital	Egypt	Arabic	97%
Knee ICOPQ	Alageel et al. 2020 [25]	90		51.1				orthopaedic surgery clinic			
KOOS-PF	Ateef 2020 [33]	95	Mean 49.75 SD 9.87 Range 40–80	0	PFPS		Anterior knee pain	Outpatient departments	KSA	Arabic	88.4%
OKS	Bodor et al. 2020 [34]	100	Mean: 62 SD: 11.3				TKR		KSA	Arabic	
OAQoL	Al-Ajmi and Al-Ghamdi 2021 [32]	59	Mean: 48.4 SD: 11.3	47.5%	OA		OA		KSA	Arabic	100%
Kujala score	Haddad et al. 2021 [41]	94	Mean 43.67 SD 14.46	70.2%	PFPS		Anterior knee pain	Orthopedic OP clinic	Jordan	Arabic	70.1%
ACL-RSI	ACL-RSI Alzhrani et al. 2022 [26]	60	Mean 11.22 SD 3.84	0%	ACL Reconstruction	Mean 11.22 SD 3.84	ACL injury	Online mode Via Google Forms	KSA	Arabic	100%
TAS	Alzhrani et al. 2022 [27]	75	Mean 32.31 SD 7.28	0%	ACL Reconstruction	>3 month Post ACL Reconstruction	ACL injury	Online mode Via SurveyMonkey	KSA	Arabic	100%
KOOS-PF-F	Alzhrani et al. 2022 [35]	105	Mean 51.62 SD 8.49 Range 34–66	100%	PFPS	NR	Anterior knee pain	PT OPD, University hospital	KSA	Arabic	87.6%

*—Cross-sectional study design (exploratory research design), Western Ontario and McMaster Universities Osteoarthritis Index (WOMAC), knee injury and osteoarthritis outcome score (KOOS), knee outcome survey- activities of daily living scale (KOS-ADLS), Oxford knee score (OKS), anterior knee pain scale, osteoarthritis of knee and hip health-related quality of life (OAKHQoL) scale, Lysholm knee score (LKS), international documentation committee subjective knee form (IKDC), intermittent and constant osteoarthritis pain (ICOAP) questionnaire, Kujala patellofemoral pain scoring system (PFPSS), anterior knee pain scale (AKPS), and osteoarthritis quality of life questionnaire (OAQoL), Tegner activity scale (TAS), short version of anterior cruciate ligament–return to sport after injury scale (ACL-RSI), knee injury and osteoarthritis outcome score patellofemoral questionnaire for females (KOOS-PF-F), patient-reported outcome measures (PROMS). OPD: outpatient department, PT: physical therapy, NR: not reported, ACL: anterior cruciate ligament.

**Table 3:** Information to extract on the interpretability of PROMs.

PROM (ref)	Distribution of Scores in the Study Population	Percentage of Missing Items and Percentage of Missing Total Scores	Floor and Ceiling Effects	Scores and Change Scores Available for Relevant (sub) Groups	Minimal Important Change (MIC) or Minimal Important Difference (MID)
Reduced WOMAC (Alghadir et al. 2016) [28]	Pain 1–16 Function 1–27 Total 3–43	NR	NR	NR	Pain 3.80 Function 5.24 Total 8.15
KOOS (Alfadhel et al. 2018) [43]	Pin 45.6 ± 18.7 Symptom 52.9 ± 21 ADL 47.4 ± 20.1 Sports 17.7 ± 18.9 Knee QOL 31 ± 17	?	Floor Pain 0.7% Sports 26.5% Knee QOL 3.7% Ceiling Symptoms 0.7%	NR	Pain 13.91 Symptoms 14.25 ADL 13.46 Sports 14.56 Knee QOL 12.57
KOS-ADLS (Algarni et al. 2017) [30]	NR	NR	NR	NR	NR
Oxford Knee Score (OKS) (Alghadir et al. 2017) [29]	Function 12–59	NR	Floor 2.1% Ceiling 1%	NR	Function 6.2
Anterior Knee Pain Scale (Alshehri et al. 2017) [31]	Total 59.3 ± 17.3	NR	0% each	NR	NR
Moroccan WOMAC (Faik et al. 2008) [37]	Pain 10.7 ± 3.9 Stiffness 4.45 ± 1.95 ADL 38.48 ± 11.65 Total 53.59 ± 16.32	NR	NR	NR	NR
Moroccan OAKHQoL (Serhier et al. 2012) [36]	PA 42.5 ± 21.6 Mental health 52.8 ± 20.7 Pain 45.0 ± 27.8 Social support 59.4 ± 24.0 Social function 60.8 ± 27.5	Reported Average 2.1% Range 0–52%	Reported Floor (individual item range) 2.6–65.6% Ceiling (individual item range) 7.7–56.5%	Reported Age, sex, and joints involved	NR
Sfax Modif WOMAC (Guermazi et al. 2004) [14]	Pain 3–19 Stiffness 0–8 Function 2–27	Reported 3 questions (function subscale) removed	Ceiling 0% Floor 0%	NR	NR
KOOS (Almangoush et al. 2013) [39]	Pain 3–72 Symptoms 4–64 ADL 0–62 Sport 5–100 QOL 3–72	0.21% of all answered items	Ceiling- 3.1% in ADL subscale Floor- 1.6% in sport subscale	NR	NR
KOS-ADLS (Bouzubar et al., 2018) [42]	Total 50.4 ± 18.1	NR	Ceiling 0% Floor 0%	NR	MID 14% MIC 8.7
Kujala PFPSS (Hamdan et al. 2019) [40]	62.38 ± 17.78 to 64.02 ± 18.47	NR	NR	NR	NR
OKS, LKS, IKDC (Ahmed et al. 2019) [38]	NR	NR	NR	NR	NR
Knee ICOPQ (Alageel et al. 2020) [25]	NR	NR	NR	NR	NR
KOOS-PF (Ateef 2020) [33]	NR	NR	NR	NR	NR
OKS (Bodor et al. 2020) [34]	NR	NR	NR	NR	NR
OAQoL (Al-Ajmi and Al-Ghamdi 2021) [32]	35.63 ± 12.25		Floor–4,7% to 13.9%; ceiling–2.2% to 13.4%		MDC: 16.91%
Kujala score (Haddad et al. 2021) [41]	63.91 ± 16.32 to 66.52 ± 17.50	NR	NR	NR	NR
ACL-RSI (Alzhrani et al. 2022) [26]	29.72 ± 9.91	NR	Floor—5%; Ceiling—0%;	NR	MDC_Individual_: 20.08; MDC_Group_: 3.44
TAS (Alzhrani et al., 2022) [27]	4.60 ± 2.75	NR	Floor—0%; ceiling 2.7.6%	NR	MDC_Individual_: 2.39; MDC_Group_: 0.41
KOOS-PF-F (Alzhrani et al., 2022) [35]	29.72 ± 9.91	NR	Floor—0.9% to 13.9%; ceiling—3.8% to 13.6%	NR	MDC: 22.96%

Western Ontario and McMaster Universities Osteoarthritis Index (WOMAC), knee injury and osteoarthritis outcome score (KOOS), knee outcome survey- activities of daily living scale (KOS-ADLS), Oxford knee score (OKS), anterior knee pain scale, osteoarthritis of knee and hip health-related quality of life (OAKHQoL) scale, Lysholm knee score (LKS), international documentation committee subjective knee form (IKDC), intermittent and constant osteoarthritis pain (ICOAP) questionnaire, Kujala patellofemoral pain scoring system (PFPSS), anterior knee pain scale (AKPS), and osteoarthritis quality of life questionnaire (OAQoL). Tegner activity scale (TAS), short version of anterior cruciate ligament–return to sport after injury scale (ACL-RSI), knee injury and osteoarthritis outcome score patellofemoral questionnaire for females (KOOS-PF-F), patient-reported outcome measures (PROMS).

**Table 4:** Results of studies on measurement of PROMs.

PROM (Ref)	Country (Language) in which the PROM Was Evaluated	Modifications	Internal Consistency	Construct Validity	Reproducibility	Floor/Ceiling Effect (%)	Responsiveness	Quality COSMIN
Reduced WOMAC (Alghadir et al. 2016) [28]	Saudi Arabia (Arabic)	Cultural adaptations	?	NR	Pain 0.89 Function 0.90 Total 0.91	NR	NR	?
KOOS (Alfadhel et al. 2018) [43]	Saudi Arabia (Arabic)	Cultural adaptations	Pain 0.87 Sym 0.91 ADL 0.88 Sport 0.92 QOL 0.90	NR	Pain 0.93 Symptom 0.94 ADL 0.94 Sport 0.92 Knee QOL 0.93	Floor Pain 0.7% Sports 26.5% Knee QOL 3.7% Ceiling Symptoms 0.7%	NR	?
KOS-ADLS (Algarni et al. 2017) [30]	Saudi Arabia (Arabic)	Cultural adaptations	Total 0.902	NR	NR	NR	NR	?
Oxford Knee Score (OKS) (Alghadir et al. 2017) [29]	Saudi Arabia (Arabic)	Cultural adaptations	Total 0.98	NR	Total 0.973	Floor 2.1% Ceiling 1%	NR	?
Anterior Knee Pain Scale (Alshehri et al. 2017) [31]	Saudi Arabia	Cultural adaptations	Total 0.81	NR	Total 0.96	0% each	NR	?
Moroccan WOMAC (Faik et al. 2008) [37]	Morocco (Arabic- Moroccan dialect)	Cultural adaptations	Pain 0.76 Stiff 0.76 ADL 0.90 Total 0.92	NR	Pain 0.80 Stiffness’ 0.77 ADL 0.89 Total 0.91	NR	NR	?
Moroccan OAKHQoL (Serhier et al. 2012) [36]	Morocco (Arabic- Moroccan dialect)	Cultural adaptations	PA 0.93 Mental heal 0.84 Pain 0.88 Social sup 0.50 Social function 0.60	Age- no diff except PA Sex- no differ Joint involves- no differ	PA Inter 0.90 Intra 0.83 Mental health Inter 0.83 Intra 0.65 Pain Inter 0.81 Intra 0.70 Social support Inter 0.64 Intra 0.71 Social function Inter 0.58 Intra 0.54	Reported Floor (individual item range) 2.6–65.6% Ceiling (individual item range) 7.7–56.5%	NR	?
Sfax Modif WOMAC (Guermazi et al. 2004) [14]	Tunisia (North African dialect)	Cultural adaptations	NR	NR	Pain 0.84 Stiffness 0.84 Function 0.92	Ceiling 0% Floor 0%	NR	?
KOOS (Almangoush et al. 2013) [39]	Arabic	Cultural adaptations	Pain 0.92 Symptom 0.82 ADL 0.95 Sport 0.91 QOL 0.80	NR	Pain 0.954 Symptom 0.931 ADL 0.957 Sport 0.941 QOL 0.875	Ceiling- 3.1% in ADL subscale Floor- 1.6% in sport subscale	NR	?
KOS-ADLS (Bouzubar et al., 2018) [42]	Arabic	Cultural adaptations	Total 0.97	NR	Total 0.97	Ceiling 0% Floor 0%	ES 1.12 Improvement in 86.7% AUC 0.73 Functional improvement 14%	?
Kujala PFPSS (Hamdan et al. 2019) [40]	Arabic	Translations	Total: 0.824		Total: ICC = 0.948 (0.923–0.965)	NR	Improvement in 47.4%	?
LKS (Ahmed et al. 2019) [38]	Arabic	Cultural adaptations	Total: 0.9	KOOS: 0.7	Total: ICC = 0.8	NR	NR	?
OKS (Ahmed et al. 2019) [38]	Arabic	Cultural adaptations	Total: 0.9	KOOS: 0.913	Total: ICC = 0.85	NR	NR	?
IKDC (Ahmed et al. 2019) [38]	Arabic	Cultural adaptations	Total: 0.89	KOOS: 0.58	Total: ICC = 0.95	NR	NR	?
Knee ICOPQ (Alageel et al. 2020) [25]	Arabic	Cultural adaptations	Total: 0.88	KOOS: 0.235	Total: ICC = 0.841	NR	NR	?
KOOS-PF (Ateef 2020) [33]	Arabic	Cultural adaptations	Total: 0.81	−0.568	Total: ICC = 0.959 (0.855–0.965)	Ceiling: 2.2–13.4% Floor: 4.7–13.9%	NR	+
OKS (Bodor et al. 2020) [34]	Arabic	Cultural adaptations	Total: 0.85	KOOS-Ar: r_s_ = 0.73	Total: r_s_ = 0.94	Ceiling 0% Floor 0%	3.09	?
OAQoL (Al-Ajmi and Al-Ghamdi 2021) [32]	Saudi Arabia (Arabic)	Cultural adaptations	Total: 0.93	NR	Total: ICC = 0.93	NR	NR	?
Kujala score (Haddad et al., 2021) [41]	Arabic	Cultural adaptations	Total: 0.806	NR	Total: ICC = 0.806 (0.742–0.859)	NR	Improvement in 45.7%	?
ACL-RSI (Alzhrani et al., 2022) [26]	Arabic	Cultural adaptations	Total: 0.734	IKDC-Ar: r_s_ = 0.515; KOOS-Ar: r_s_ = 0.542	Total: ICC = 0.871 (0.743–0.935)	Ceiling—0%; Floor—5%	NR	-
TAS (Alzhrani et al., 2022) [27]	Arabic	Cultural adaptations	NR	IKDC-Ar: r_s_ = 0.476; KOOS-Ar: r_s_ = 0.469	Total: ICC = 0.836 (0.687–0.914)	Ceiling—2.7.6%; Floor—0%	NR	-
KOOS-PF-F (Alzhrani et al., 2022) [35]	Arabic	Cultural adaptations	Total: 0.93	−0.783	Total: ICC = 0.893 (0.889–0.970)	Ceiling: 0.9%–13.9% Floor: 3.8%–13.6%	NR	?

Western Ontario and McMaster Universities Osteoarthritis Index (WOMAC), knee injury and osteoarthritis outcome score (KOOS), knee outcome survey- activities of daily living scale (KOS-ADLS), Oxford knee score (OKS), anterior knee pain scale, osteoarthritis of knee and hip health-related quality of life (OAKHQoL) scale, Lysholm knee score (LKS), international documentation committee subjective knee form (IKDC), intermittent and constant osteoarthritis pain (ICOAP) questionnaire, Kujala patellofemoral pain scoring system (PFPSS), anterior knee pain scale (AKPS), and osteoarthritis quality of life questionnaire (OAQoL). Tegner activity scale (TAS), short version of anterior cruciate ligament–return to sport after injury scale (ACL-RSI), knee injury and osteoarthritis outcome score patellofemoral questionnaire for females (KOOS-PF-F), patient-reported outcome measures (PROMS).

**Table 5:** The methodological quality of PROMs related to knee joint according to the COSMIN scale.

PROM (ref)	Structural Validity	Internal Consistency	Reliability	MEASUREMENT Error	Hypothesis Testing for Construct Validity	Cross-Cultural Validity	Criterion Validity	RESPONSIVENESS	Final
Reduced WOMAC (Alghadir et al. 2016) [28]	+	+	+	+	?	?	?	?	?
KOOS (Alfadhel et al. 2018) [43]	+	+	+	+	?	?	?	?	?
KOS-ADLS (Algarni et al. 2017) [30]	+	+	+	+	+	+	+	+	-
Oxford Knee Score (OKS) (Alghadir et al. 2017) [29]	+	+	+	+	?	?	?	?	?
Anterior Knee Pain Scale (Alshehri et al. 2017) [31]	+	+	+	?	?	?	?	?	?
Moroccan WOMAC (Faik et al. 2008) [37]	+	+	+	+	+	?	?	?	?
Moroccan OAKHQoL (Serhier et al. 2012) [36]	+	+	+	-	+	?	?	?	?
Sfax Modif WOMAC (Guermazi et al. 2004) [14]	+	+	+	+	+	+	+	+	-
KOOS (Almangoush et al. 2013) [39]	+	+	+	+	+	+	+	+	-
KOS-ADLS (Bouzubar et al. 2018) [42]	+	+	+	+	?	?	?	+	?
Kujala PFPSS (Hamdan et al. 2019) [40]	+	+	+	-	?	?	?	?	?
LKS (Ahmed et al. 2019) [38]	+	+	+	-	?	?	?	?	?
OKS (Ahmed et al. 2019) [38]	+	+	+	-	?	?	?	?	?
IKDC (Ahmed et al. 2019) [38]	+	+	+	-	+	?	?	?	?
Knee ICOPQ (Alageel et al. 2020) [25]	+	+	+	-	+	?	?	?	?
KOOS-PF (Ateef 2020) [33]	+	+	+	+	+	+	-	+	-
OKS (Bodor et al. 2020) [34]	+	+	+	?	?	?	?	?	?
OAQoL (Al-Ajmi and Al-Ghamdi 2021) [32]	+	+	+	?	?	?	?	?	?
Kujala score (Haddad et al., 2021) [41]	-	+	?	-	?	?	?	?	-
ACL-RSI (Alzhrani et al., 2022) [26]	-	+	+	+	+	?	-	?	-
TAS (Alzhrani et al., 2022) [27]	-	?	+	+	+	?	-	?	-
KOOS-PF-F (Alzhrani et al., 2022) [35]	+	+	+	+	+	+	+	?	?

Western Ontario and McMaster Universities Osteoarthritis Index (WOMAC), knee injury and osteoarthritis outcome score (KOOS), knee outcome survey- activities of daily living scale (KOS-ADLS), Oxford knee score (OKS), anterior knee pain scale, osteoarthritis of knee and hip health-related quality of life (OAKHQoL) scale, Lysholm knee score (LKS), international documentation committee subjective knee form (IKDC), intermittent and constant osteoarthritis pain (ICOAP) questionnaire, Kujala patellofemoral pain scoring system (PFPSS), anterior knee pain scale (AKPS), and osteoarthritis quality of life questionnaire (OAQoL). COSMIN (COnsensus-based Standards for the selection of health Measurement Instruments), Tegner activity scale (TAS), short version of anterior cruciate ligament–return to sport after injury scale (ACL-RSI), knee injury and osteoarthritis outcome score patellofemoral questionnaire for females (KOOS-PF-F), patient-reported outcome measures (PROMS). (from high-to-low: ‘“+” = sufficient’, ‘“-” = insufficient’, ‘“?” = indeterminate’).

### 3.6. Reporting Risk of Bias across Studies

Knee OA was the primary diagnosis of all studies except two, i.e., patellofemoral pain [31], ACL, meniscal, and combined injuries [39]. All studies included more than a hundred patients except six [26,27,29,31,37,41] with 40 patients as lowest [31]. The mean age of the included studies was greater than 50 years in all studies except three [31,39,42]. All studies included both genders, except three [26,27,29], where all patients were males, and one study [35] recruited only females.

### 3.7. Summary of Evidence

Twenty validated tools have been compiled in this systematic review and assessed for their methodological quality of psychometric properties of patient-reported outcome measures (PROMs) of knee-related disease-specific questionnaires in Arabic. Of them, 12 were validated in the Saudi Arabian version of Arabic, and the remaining eight were other than the Saudi Arabian versions.

The internal consistency (IC) of all the included studies had obtained a Cronbach’s α value between 0.7 and 0.9, the total Cronbach’s α value of Oxford knee score (OKS) by Alghadir et al. 2017 was the highest scored value [30], with an excellent consistency between the items, and the patients would have found a good flow of disease symptoms in osteoarthritis (OA) participants than the rest of the included studies [25,28,30,31,32,33,34,43].

The reproducibility of all the included studies, with a considerable time gap, yielded a good test-retest reliability property of more than 0.8, which is the minimum required measurement value as per the psychometric analysis; 0.841 as the lowest by Alageel M et al. [23], and the rest of the eight studies were above the required measurement values, with a good to excellent reliability [23,24,25,26,27,28,29,30,31]. Furthermore, of different domains/subscales of all the included studies, the Oxford knee score (OKS) by Alghadir et al. 2017 obtained a total ICC of 0.973, though with a good recall period of one-week duration [27], representing a promising property.

All the studies conducted in Saudi Arabia have shown structural validity; however, most of the studies did not fully (intermediate only) report the hypothesis testing for construct validity, except knee ICOPQ by Alageel et al. 2020; KOOS-PF by Ateef 2020; OKS by Bodor et al. 2020 [25,33,34]. The Arabic OKS version pain item subscale was associated strongly with the Arabic KOOS pain subscale (rs = 0.73), as the pain threshold in TKR awaiting patients was considered high and correlated strongly with the Arabic KOOS pain subscale, where the correlation coefficient above 0.70 was considered strong [24].

Most of the included studies were not fully reported in cross-cultural validity, except for two studies, KOS-ADLS by Algarni et al. 2017 [30] and KOOS-PF by Ateef 2020 [30]. In the later study, the cross-cultural validity was very well reported by adapting the religious activity, such as prayers, where most of the items of KOOS-PF were analogues to the prayer activities; the patients with extreme symptoms would be able to appreciate the symptoms of the disease during such activities, fulfilling the meaning of true cross-cultural adaptation and psychometric validation of the adapted questionnaire [33]. A study by Algarni et al. 2017 has tried to justify the cross-cultural adaptation by adjuring the cultural background with a kneeling construct, which the Muslim populace could comprehend easily [30].

#### 3.7.1. Responsiveness to Treatment Properly

None of the included studies conducted treatment affect outcomes, compared to baseline symptoms [9,14,25,26,27,28,30,31,32,33,34,35,36,37,38,39,40,41,42,43]. However, the four studies [14,30,35,39], aside from fulfilling the progress response to the given interventions as process to validate the responsiveness psychometric property, achieve other properties of COSMIN; thereby accomplishing all the properties, a high methodological quality of a questionnaire according to the COSMIN (COnsensus-based Standards for the selection of health Measurement Instruments) checklist [22].

#### 3.7.2. Minimal Important Change (MIC) or Minimal Important Difference (MID)

An Arabic tool reduced WOMAC by Alghadir et al. 2016 has shown that the total minimal important change (MIC) or minimal important difference (MID) score was 8.13, indicating a good response to treatment outcomes [28].

#### 3.7.3. Floor/Ceiling Effect (%)

Eight studies from Saudi Arabia have reported the floor/ceiling effect in percentage [8,26,27,29,31,34,35,43]. However, few reported floor/ceiling zero percent effect, indicating no negative/positive responses of the measured construct/health [31,33], and the rest were within the acceptable limits.

Among the PROMs, the KOS-ADLS seems to be a better option as it has fewer items [42], and can be used in various conditions. In addition, it has been validated in both clinical and post-surgical conditions. All measurements based on COSMIN guidelines were evaluated for KOS-ADLS, and had methodologically high-quality ratings [30]. Both WOMAC and KOOS could be used in knee OA patients [14,39], but inconsistencies found in WOMAC [14] lead to the selection of KOOS to evaluate knee OA [39].

Even though WOMAC was validated in three countries, its content was different between studies. For example, Sfax WOMAC [14] removed some items from the function subscale, whereas reduced WOMAC [28] had a low number of items in the subscale. The original complete WOMAC was used by Faik et al. in the Moroccan Arabic dialect. KOOS [37], an extension of WOMAC, was used in two studies without mentioning such difficulties. OKS [38] and KOOS-PF [33] were not validated in females before 2022 [35]. But later, the Arabic translation of OKS overcame this shortcoming by recruiting 45% of females during the translation and validation process, and in KOOD-PF-F [35]. Different dialects among the Arabic-speaking population are another important limitation in selecting knee-related PROMs. 

## 4. Discussion

This is the first study to conduct a systematic review of studies based on COSMIN guidelines to evaluate the methodological quality of psychometric properties of different Arabic knee-related outcome measures. Among the three PROMs (WOMAC, KOS-ADLS, and KOOS-PF-F) [14,30,35,39], with two or more studies, KOS-ADLS seems a better option because it has fewer items [38] and can be used in various conditions. In addition, it has been validated in both clinical and post-surgical conditions. All measurement properties are evaluated for KOS-ADLS and have methodologically high-quality studies [30]. WOMAC [14] and KOOS [39] could be used in knee OA patients, but inconsistencies found in WOMAC lead to the selection of KOOS to evaluate knee OA. In addition to this, for evaluating PFPS among females, KOOS-PF-F [35] could be used. None of the included studies [9,14,25,26,27,28,30,31,32,33,34,35,36,37,38,39,40,41,42,43] have the responsiveness measurement property of COSMIN. However, the four studies [14,30,35,39], except for fulfilling measurement property, responsiveness, have other methodological qualities as recommended by COSMIN [24]. 

Overall, our review points to a scarcity of evidence of sufficient psychometric properties of knee-related outcome measures translated, cross-culturally adapted, and validated in Arabic. However, we wish to highlight a few key points that were borne in mind while decoding our outcomes. The COSMIN tool comprises categories, with essential arbitrary scores chosen as cut-offs to discriminate between adequate and inadequate measurement properties. Occasionally, the statistical outcomes leading to a negative rating consist of a proximate score to the acceptable positive rating. This point mimics methodological quality ratings, the ‘worst score counts’ algorithm reported by COSMIN. It was measured on three points rating scale (i.e., from high-to-low: ‘“+” = sufficient’, ‘“−” = insufficient’, ‘“?” = indeterminate’) [23]. This signifies the terminal rating of methodological quality described by the minimum score obtained for that measurement property; thus, a single flaw can guide to a rating of ‘insufficient’ when it is alternatively rated as ‘sufficient’. We employed a similar rule while rating the adequacy of psychometric properties of knee-related outcome measures translated, cross-culturally adapted, and validated in Arabic, where data for various subscales were provided: one sub-optimal score was sufficient to yield a negative rating of the adequacy of that particular property. The inference of these key points provides a glance at our findings, which may lead to an underestimation of the adequacy of measurement properties and methodological quality of the evidence.

However, including studies specifically not focused on examining the psychometric properties would have boosted the risk of bias, paving the path to an unwieldy number of studies for review, and becoming a more challenging task for future researchers to reproduce our review. Yet, we agree that by adapting COSMIN, we have undertaken a rigorous approach for the selection and rating. Furthermore, we believe that this review has collectively acknowledged the state of evidence on psychometric properties for individual PROMs.

The COSMIN initiative focuses on developing new and updating the existing methodology criteria, based on broad consensus. The COSMIN criteria have been introduced recently, focusing on biomedical healthcare and research, and measuring constructs such as health-related quality of life, symptom status, or functional status [21,23,24]. Later, the methodology extended its scope to systematic reviews in other healthcare contexts, like pediatric populations [45,46] and patients with fibromyalgia [47]. Considering this review, it should be taken in mind that many studies on psychometric properties of knee-related outcome measures translated, cross-culturally adapted, and validated in the Arabic language were accomplished before the publication of COSMIN criteria, which signifies that authors of previous studies were not aware of these criteria and/or did not use them in their research. Also, it has not yet been understood whether these standards generally apply to all types of PROMs.

### Limitations

The scoring system adopted by COSMIN is the limitation of the systematic review of psychometric properties of knee-related outcome measures translated, cross-culturally adapted, and validated in Arabic. We have the issue with counting the lowest score in assessing the methodological quality of PROMs included in this review. Therefore, we have rated according to COSMIN criteria; the overall score is ‘“?” = indeterminate’ even though the particular PROM has ‘“+” = sufficient’ on all requirements except one criterion.

Another concern regarding the heterogeneity of measurement properties reported in the included PROMs is that most of the study does not provide the same amount of required information as recommended by COSMIN. Also, the heterogeneity of patient conditions was used in the review as few studies were used to record PROM post-surgery as in ACLR, while few were in regular rehabilitation follow-ups such as knee OA. Last, we could not get a clear idea whether the psychometric properties of knee-related outcome measures translated, cross-culturally adapted, and validated in the Arabic language used in this review were not performed as recommended by COSMIN, or not reported as recommended by COSMIN.

Studies in our review report revealed a range of different statistics across the measurement properties. Also, there was no valid and reliable method to check the publication bias and researcher bias towards publishing positive results. As a result, it is possible that our review overestimates the adequacy of psychometric properties across measures since there may be unpublished data showcasing the negative results.

Finally, we end this discussion by conveying those four scales, WOMAC [14], KOOS [39], KOS-ADLS [30], and KOOS-PF-F [35], have methodologically high-quality grades based on COSMIN guidelines for evaluating PROMs, except the property of responsiveness. However, the above limitations should be considered before their clinical implications.

## 5. Conclusions

Current evidence among the included studies reflect that all knee Arabic PROMs are reliable instruments to evaluate knee symptoms/function. Among them, KOS-ADLS is the best PROM to be used in various knee conditions with high-quality evidence, followed by KOOS, WOMAC could be utilized in knee OA patients, and KOOS-PF-F among females with PFPS.

## Figures and Tables

**Table 1:** Characteristics of the included PROMs.

PROM	Target Population	Mode of Administration (e.g., self-Report, Interview-Based, Parent/Proxy Report, etc.)	Recall Period	(Sub)Scale (s) (Number of Items)	Response Options	Range of Scores/Scoring	Original Language	Available Translations
Reduced WOMAC (Alghadir et al. 2016) [28]	Knee OA	Self-report	48 h	Pain (5); Function (7)	0–4	Pain 0–20 Function 0–28 Overall 0–48	English	Arabic (Saudi)
KOOS (Alfadhel et al. 2018) [43]	Knee OA	Self-report	1 week	Pain (9); Symptom (7); ADL (17); Sports (5); Knee QOL (4)	0–4	0–100 for each scale where a higher score indicates better health	English	Arabic (Saudi)
KOS-ADLS (Algarni et al. 2017) [30]	Various Knee complaints (Knee OA 88.2%)	Self-report	Within 1 week	Symptom (6); Function (8)	0–5	0–100	English	Arabic (Saudi)
Oxford Knee Score (OKS) (Alghadir et al. 2017) [29]	Knee OA	Self-report	Within 1 week	Function (12)	0–4	0–48	English	Arabic (Saudi)
Anterior Knee Pain Scale (Alshehri et al. 2017) [31]	Patellofemoral Pain	Self-report	2–3 days	Symptom and Function (13)	Varying	0–100	English	Arabic (Saudi)
Moroccan WOMAC (Faik et al. 2008) [37]	Knee OA	Self-report	1–2 days	Pain (5); Stiffness (2); ADL (17);	0–4	Pain 0–20 Stiffness 0–8 ADL 0–68 Total 0–96	English	Arabic (Moroccan)
Moroccan OAKHQoL (Serhier et al. 2012) [36]	Knee and Hip OA	Self-report	3–10 days	PA (16); Mental Health (13); Pain (4); Social support (4); Social function (3); 3 items;	Individual items 1–10	Subscales 0–100 100 mean best QOL	French	Arabic (Moroccan)
Sfax Modif WOMAC (Guermazi et al. 2004) [14]	Knee OA	Interviewer	24 h	Pain (5); Stiffness (2); Function (9);	0–4	Pain 0–20 Stiffness 0–8 Function 0–36	English	Arabic (Tunisia)
KOOS (Almangoush et al. 2013) [39]	ACL, Meniscal, and combined injury	Self-report	1 week	Pain (9); Symptom (7); ADL (17); Sport (5); QOL (4)	0–4	0–100 in which 100 means no Knee problem	English	Arabic (Egypt)
KOS-ADLS (Bouzubar et al., 2018) [42]	Various Knee conditions (63%) and post-surgery (37%)- Knee OA 35%	Self-report	2–4 days	Symptom (6); Function (8)	0–5	0–100 in which 100 means perfect health	English	Arabic (Kuwait)
Kujala PFPSS (Hamdan et al. 2019) [40]	Patellofemoral pain syndrome	Self-report	2 Weeks	The severity of symptoms (13 factors)	0–10	0–100 in which 100 means good Knee function	English	Arabic (Jordan)
OKS, LKS, IKDC (Ahmed et al. 2019) [38]	ACL tear, meniscus tear, and knee osteoarthritis	Self-report	15 days	Instability (25); Pain (25); Catching (15); Stair climbing (10); Swelling (10); Support (5); Squatting (5); Limping (5)	0–5/10/15/25	0–100 in which 100 means symptoms	English	Arabic (Egypt)
Knee ICOPQ (Alageel et al. 2020) [25]								Arabic (Saudi)
KOOS-PF (Ateef 2020) [33]	Patellofemoral pain syndrome	Self-report	48 h	Symptom (1); Pain (9); QoL (1)	0–4	0–100 in which 100 means no Knee problem	English	Arabic (Saudi)
OKS (Bodor et al. 2020) [34]	TKA	Self-report	7–10 days					Arabic (Saudi)
OAQoL (Al-Ajmi and Al-Ghamdi 2021) [32]	Osteoarthritis	Self-report					English	Arabic (Saudi)
Kujala score (Haddad et al., 2021) [41]	Patellofemoral pain syndrome	Self-report	2 Weeks	The severity of symptoms (13 factors)	0–10	0–100 in which 100 means good Knee function	English	Arabic (Jordan)
ACL-RSI (Alzhrani et al., 2022) [26]	ACL Reconstruction	Self-report	<1–month	Emotions; Confidence; Risk evaluation;		1–100	English	Arabic (Saudi)
TAS (Alzhrani et al., 2022) [27]	ACL Reconstruction	Self-report	<1–month	Sedentary jobs to heavy manual (1–5); Recreational to competitive sports (6–9); Elite sports (10	Varying	0–100 in which 100 means good Knee function	English	Arabic (Saudi)
KOOS-PF-F (Alzhrani et al., 2022) [35]	Patellofemoral pain syndrome	Self-report	48 h	Symptom (1); Pain (9); QoL (1)	0–4	0–100 in which 100 means no Knee problem	English	Arabic (Saudi)

Western Ontario and McMaster Universities Osteoarthritis Index (WOMAC), knee injury and osteoarthritis outcome score (KOOS), knee outcome survey- activities of daily living scale (KOS-ADLS), Oxford knee score (OKS), anterior knee pain scale, osteoarthritis of knee and hip health-related quality of life (OAKHQoL) scale, Lysholm knee score (LKS), international documentation committee subjective knee form (IKDC), intermittent and constant osteoarthritis pain (ICOAP) questionnaire, Kujala patellofemoral pain scoring system (PFPS), anterior knee pain scale (AKPS) and osteoarthritis quality of life questionnaire (OAQoL). Tegner activity scale (TAS), short version of anterior cruciate ligament–return to sport after injury scale (ACL-RSI), knee injury and osteoarthritis outcome score patellofemoral questionnaire for females (KOOS-PF-F), patient-reported outcome measures (PROMS).

## Data Availability

Not applicable.
